# Supplementary material for: An Early Disturbance in Serotonergic Neurotransmission Contributes to the Onset of Parkinsonian Phenotypes in Drosophila melanogaster
Source: Cells. 2022 May 5;11(9):1544. doi: 10.3390/cells11091544 (PMC9105628; doi:10.3390/cells11091544)
Supplement: Supplementary file 1 [file cells-11-01544-s001.zip › cells-1659281-supplementary.pdf]

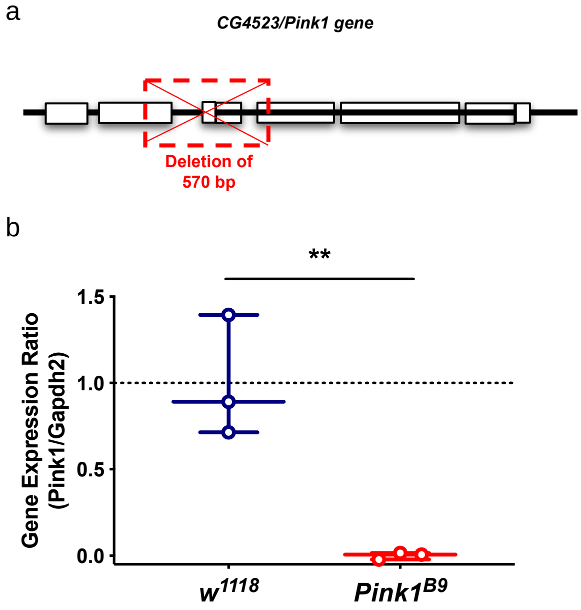

Figure S1. *Pink1[B9]* mutant fly. A schematic representation of the *Drosophila Pink1* gene: exons are indicated in boxes, and coding regions are colored black (**a**). The rectangle with dashed red line indicates the deleted region in the mutant. Quantification of RT-qPCR represented as Gene Expression Ratio of *Pink1* transcripts normalized to *Gapdh2* transcript levels (**b**). Data shown represents interquartile range with maximum and minimum range from $n = 3$ independent RT-qPCR experiments, ** indicates $p < 0.01$; Student’s t-test. Dashed black line refers to the mean of control fly.

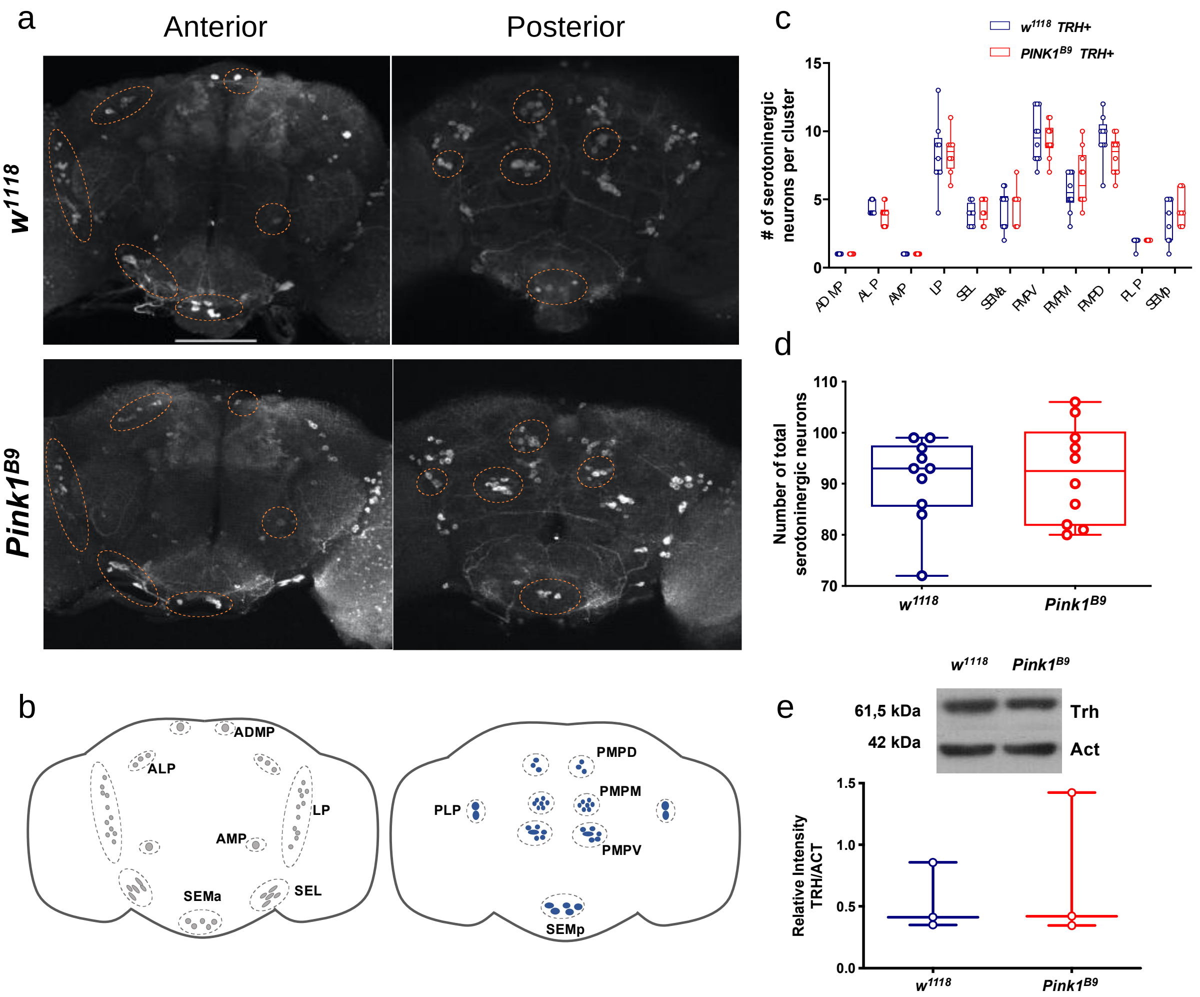

Figure S2. No changes in number of serotonergic neurons and TRH protein levels are associated with reduced 5-HT content in *Pink1*^{B9} mutant flies. Representative images of Trh+ neurons in control and *Pink1*^{B9} flies, anterior (left) and posterior (right) halves of the fly brain (**a**). The serotonergic clusters are identified in orange circles delimited with dashed lines. Schemes of the serotonergic clusters described in the literature (**b**). Quantification of the number (#) of serotonergic neurons per cluster and per hemisphere in 14-days old control (blue boxes) and *Pink1*^{B9} mutant (red boxes) flies (**c**). Quantification of the total number of Trh positive serotonergic neurons in 14-days old flies (**d**). Data in c and d are presented as interquartile range with maximum and minimum range of 10 left brain hemispheres per genotype; two-way ANOVA followed by Bonferroni's post-test indicate no significant differences in c; and student's t-test indicates no statistical differences between genotypes in d. Western blot for Trh protein in 14-17 days old *Pink1*^{B9} and *w*^{1118} control flies (**e**). As a loading control, blots for Actin (Act) are also shown. Upper panel presents a representative experiment with results for Trh (upper bands) and Act (lower bands). Lower panel shows

quantification (expressed as relative intensity) of Trh and Act bands from 3 independent experiments, each one consisting of 60 head flies. Data are presented interquartile range with maximum and minimum range. The Student's *t*-test indicates no statistical differences between mutant and control groups.

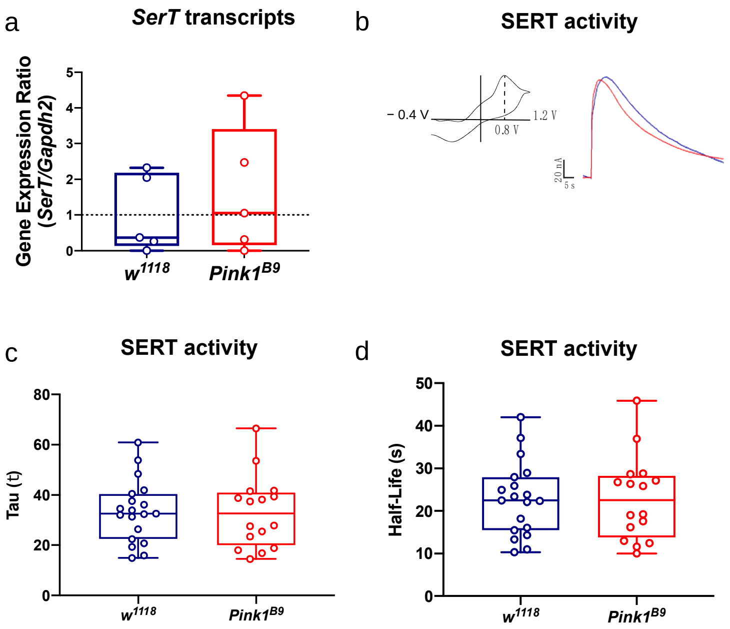

Figure S3. SerT activity in 0-3 days old flies. *SerT* transcript levels were evaluated in 3- days old w^{1118} control and $Pink1^{B9}$ mutant flies by RT-qPCR. Quantification is presented as the Gene Expression Ratio of *SerT* transcripts normalized to *Gapdh2* transcripts (**a**). Data are presented as interquartile range with maximum and minimum range. Dashed black line refers to the mean of control fly. Student's t-test indicates no statistical differences between groups. SerT activity in $Pink1^{B9}$ mutant and w^{1118} control animals evaluated by FSCV, in brains from 3-days old flies (**b**–**d**). In left panel of b, it is shown a typical voltammogram for 5-HT, while representative experiments showing 5-HT signals recorded in brains of w^{1118} control (blue line) and $Pink1^{B9}$ mutant flies (red line) are shown in right panel. *tau*, a kinetic parameter obtained from 5-HT signals recorded in fly brains from w^{1118} control and $Pink1^{B9}$ (**c**). Half-life in (s), a kinetic parameter obtained from 5-HT signals recorded in fly brains from each genotype (**d**). Data in c and d are presented as interquartile range with maximum and minimum range of 5 brains per genotype. Student's t-test indicates no statistical differences between groups.

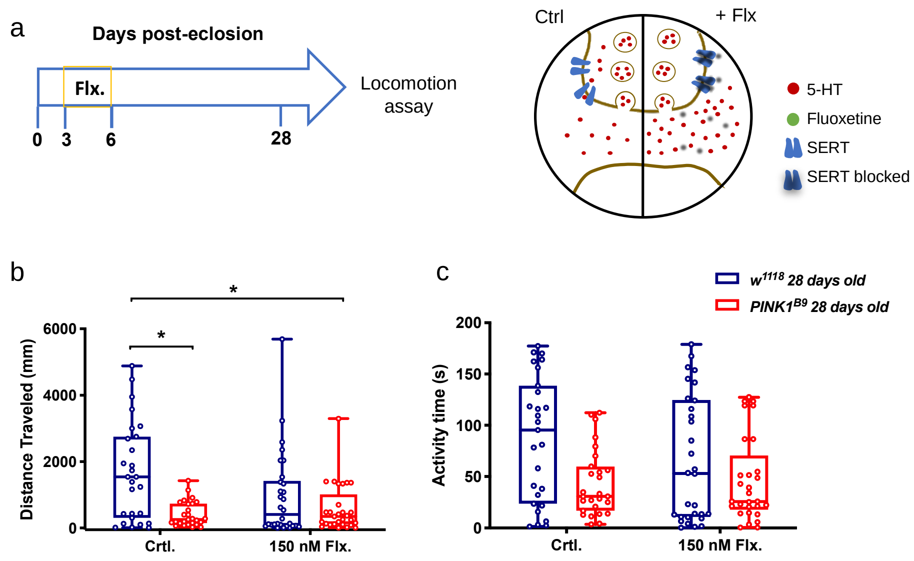

Figure S4. Effect of 150 nM fluoxetine on the locomotor behavior in 28-days old *Pink1^{B9}* mutant and control flies. (**a**). Left panel, a scheme for the timing of the fluoxetine (150 nM) treatment. Right panel, a schematic representation of the pharmacological effect of fluoxetine treatment (right, +Flx) as compared to the control situation (left, Ctrl). (**b**,**c**) Distance traveled (mm) and activity time (s), two locomotor parameters recorded in 28-days old control and *Pink1^{B9}* mutant flies treated or not with fluoxetine (150 nM Flx and Ctrl, respectively). Data was obtained from n = 27 (*w^{1118}*); 30 (*w^{1118}* + Flx); 27 (*Pink1^{B9}*); 29 (*Pink1^{B9}* + Flx) flies. Data are presented as interquartile range with maximum and minimum range. * indicates $p < 0.05$; Scheirer-Ray-Hare test followed by Dunn's post-test.

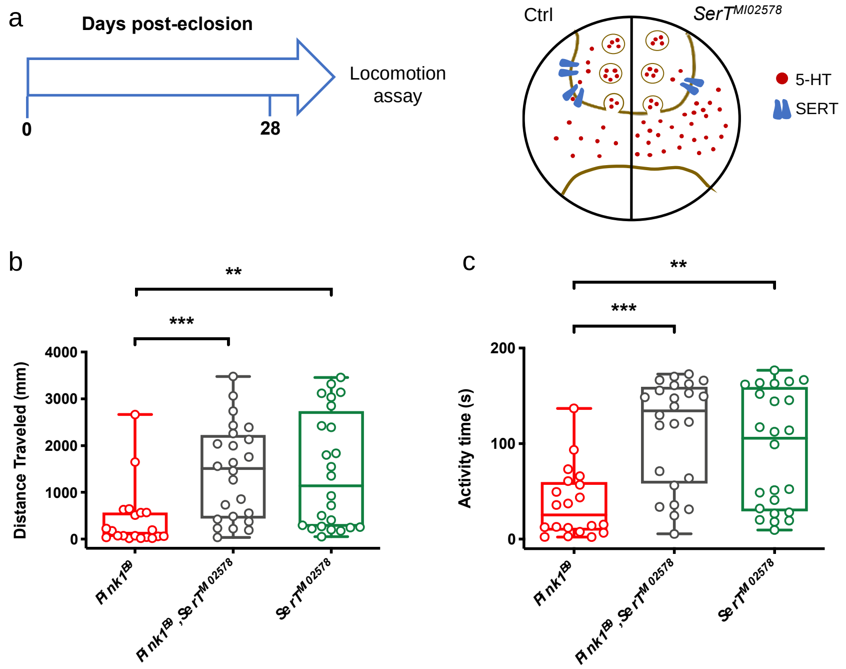

Figure S5. Effect of the genetic increase of serotonergic signaling on locomotor behavior in 28-day-old *Pink1*^{B9} mutant flies. (**a**). Left panel, a scheme for the timing of the genetic experiment during the adult stage. Right panel, a schematic representation of the serotonergic terminal exhibiting the increased serotonergic signaling as result of SerT decrease. (**b**,**c**) Distance traveled (mm) and activity time (s), two locomotor parameters recorded in 28-day-old *Pink1*^{B9},*SerT*^{MI02578} double mutant flies and their respective control flies. Data was obtained from n = 20 (*Pink1*^{B9}); 24 (*Pink1*^{B9},*SerT*^{MI02578}); 25 (*Pink1*^{B9}); 29 (*SerT*^{MI02578}) flies. Data are presented as interquartile range with maximum and minimum range. ** and *** indicate $p < 0.01$ and $p < 0.001$, respectively; Kruskal-Wallis test followed by Dunn's post hoc test.
